# Fabrication and Characterization of In Situ Synthesized SiC/Al Composites by Combustion Synthesis and Hot Press Consolidation Method

**DOI:** 10.1155/2017/9314740

**Published:** 2017-12-11

**Authors:** Hongyu Yang, Erting Dong, Bingqi Zhang, Yanyan Yuan, Shili Shu

**Affiliations:** ^1^National Demonstration Center for Experimental Materials Science and Engineering Education, Jiangsu University of Science and Technology, Zhenjiang 212003, China; ^2^Department of Materials Engineering, Henan Institute of Technology, Xinxiang 453000, China; ^3^Datang Northeast Electric Power Test And Research Institute, No. 3195 Weishan Street, Changchun 130000, China; ^4^State Key Laboratory of Luminescence and Applications, Changchun Institute of Optics, Fine Mechanics and Physics, Chinese Academy of Sciences, Changchun 130033, China

## Abstract

The in situ SiC/Al composites were fabricated in Al-Si-C systems with different Si/C mass ratios and holding time by the method of combustion synthesis and hot press consolidation. The influences of Si/C mass ratio and holding time on the phase constitution, microstructure, and hardness of the composites were investigated. The results indicate that the increase of Si/C mass ratio leads to more uniform size distribution of the SiC particles in the Al matrix. Moreover, by improving the Si/C mass ratio from 4 : 1 to 5 : 1, the maximum size of SiC particle was reduced from 4.1 *μ*m to 2.0 *μ*m. Meanwhile, the percentage of submicroparticles was increased from 22% to 63%, and the average hardness value of the composites was increased by 13%. In addition, when the holding time is set to be fifteen minutes, the Al_4_C_3_ phase did not exist in the composites because of its total reactions with Si atoms to form SiC particles, and the average hardness value was 73.8 HB.

## 1. Introduction

The SiC reinforced aluminum matrix (SiC/Al) composites have become very promising materials in the fields of semiconductor packaging, automobile, and aeronautics industry due to their high thermal conductivity and low coefficient of thermal expansion, lightweight, high strength, and wear-resistance [1–5]. For example, the SiC/Al composites which we fabricated in this work can be used for engine piston and heat sink [6, 7]. Therefore, the SiC/Al composites are actively investigated in an effort to improve their comprehensive properties [8, 9].

In recent years, several methods have been applied to fabricate the SiC/Al composites, such as stir casting [10–12], hot pressing sintering [2, 13], powder metallurgy [8], liquid pressing process [14], high pressure solidification [15], and squeeze-cast [16]. Nevertheless, in these methods the reinforcing SiC particles are usually directly added to Al matrix to form ex situ SiC/Al composites. The ex situ SiC/Al composites have several inherent disadvantages [17–19]: (I) the reinforcing SiC particles is difficult to be uniformly dispersed into the Al matrix; (II) during the incorporation of particles, interfaces between SiC and Al matrix are easy to be contaminated. In addition, cracks could appear in the interfaces due to the formation of thin oxide layers on the surfaces of the particles. To overcome these drawbacks stated above, the research on Al matrix composites is moving in two directions: one is metallic glasses replaced by typical ceramic particles reinforcements [20–22]; the other is in situ methods replaced by traditional ex situ methods.

Compared to the ex situ methods, in the in situ methods the reinforcement is synthesized through a chemical reaction among the pristine elemental materials themselves in the matrix [23, 24]. Therefore, the interfaces between reinforcement and matrix are very clean, and the bonding strength is strong. At the same time, the reinforcing particles formed by the in situ method are finer in size and uniformly distributed into the matrix [25, 26]. Nie et al. [27] fabricated the in situ SiC/Al composites by the structural evolution of TiC in Al-Si melt. They reported that the synthesis of the SiC particles occurred via the gradual reaction between the TiC and the Si atoms, and the needle-like TiAl_*x*_Si_*y*_ phase simultaneously is formed. The needle-like TiAl_*x*_Si_*y*_ phase plays a detrimental role in the mechanical properties of the composites. Du et al. fabricated the in situ SiC/Al composites by liquid-solid reaction [28] and master alloy casting method [6, 29], respectively. They reported that the formation of SiC particles was attributed to the reaction between dissolved Si atoms and Al_4_C_3_ intermediate phase, which indicates that the fabrication of in situ SiC/Al composites through gradual phase transformation mechanism was feasible. However, the formation process of the in situ SiC particles is reversible if the intermediate phase Al_4_C_3_ is as carbon source. Therefore, different reaction conditions will significantly impact on the synthesis reaction. Meanwhile, the effects of Si/C ratio and holding time on the fabrication of the in situ SiC/Al composites have not been involved in previous work. Besides, to our knowledge, the method of the combustion synthesis and hot press consolidation is another effective way for the fabrication of in situ composites [30–32]. This method takes advantage of low energy requirement, one step forming process, density, and high purity of the products.

Thus, the objectives of the present work are to fabricate the in situ SiC/Al composites in an Al-C-Si system using the method of the combustion synthesis and hot press consolidation. Meanwhile, the effects of Si/C mass ratio and holding time on the phase constitution, microstructure, and hardness of the in situ SiC/Al composites were investigated.

## 2. Experiment

**Figure 1:**
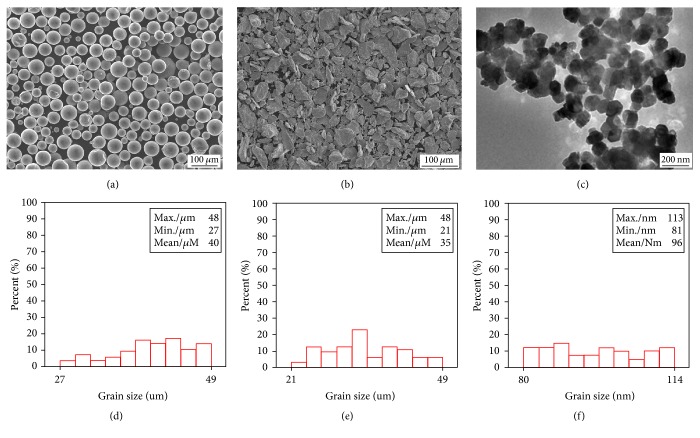
The SEM images of (a) Al, (b) Si, and (c) C (provided by Shanghai ST-Nano Science and Technology Co., Ltd.); particle size distribution of (d) Al, (e) Si, and (f) C.

In this work, commercial Al powders, Si powders, and C-black powders were used for making the powder blends. The detailed information of the raw materials is given in Table 1. The SEM images and particle size distribution are shown in Figure 1.

**Figure 2:**
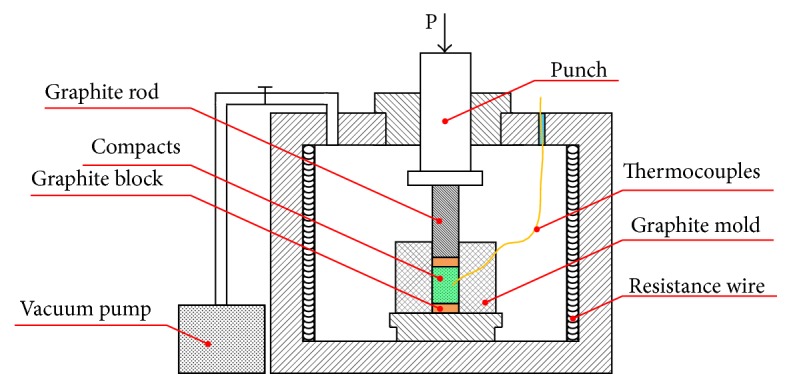
Schematic of the equipment for the combustion synthesis and hot press consolidation experiment.

The detailed fabrication process procedure is shown as follows: First, Al powders and carbon powders were mixed with different Si/C mass ratio (as shown in Table 2). The dispersion method was a dry process using ball milling. The mixtures of powders were sealed into a 500 mL volume zirconia jar together with ZrO_2_ milling balls (ball to powder mass ratio of 10). The jar was aerated with argon gas to protect the powder from excessive oxidation. The milling was carried out on a roller ball milling machine at 35 rpm for 8 h. Second, the mixtures were cold pressed into cylindrical compacts with the diameter of 28 mm and height of 30 mm. Third, the powder compact was contained in a graphite mold. And the graphite mold with powder compact was put in a self-made vacuum thermal explosion furnace as illustrated in Figure 2, in which the combustion synthesis and hot press consolidation experiment was conducted. During this process, the temperature was monitored by W5-Re26 thermocouples and the heating rate was 20°C/min. The furnace temperature was set to 950°C, and after different holding time, the pressure of 30 MPa was applied. Finally, the compact was cooled inside the furnace to room temperature. Notice that the compact was heated and cooled in a vacuum environment (≤5 × 10^−2^ Pa).

The phase constitution of the samples was investigated by X-ray diffraction (XRD, Model D/Max 2500PC, Rigaku, Tokyo, Japan) with Cu K_*α*_ (*λ* = 0.154 nm) radiation. The samples were firstly mechanical ground, then polished down to a diamond finish of 1.5 *μ*m, and then etched in a hybrid solution of 5% HCl and 95% ethanol at room temperature for 5 s for the microstructural observations. The morphology was observed by scanning electron microscopy (SEM, Model Evo18 Carl Zeiss, Oberkochen, Germany). The size measurement and distribution statistics of SiC particle were performed with the Nano Measurer software. The hardness tests were conducted on a XHB-3000 digital Brinell hardness tester according to the ASTM E10-14 standard.

## 3. Results and Discussion

### 3.1. Fabrication of In Situ SiC/Al Composites

**Figure 3:**
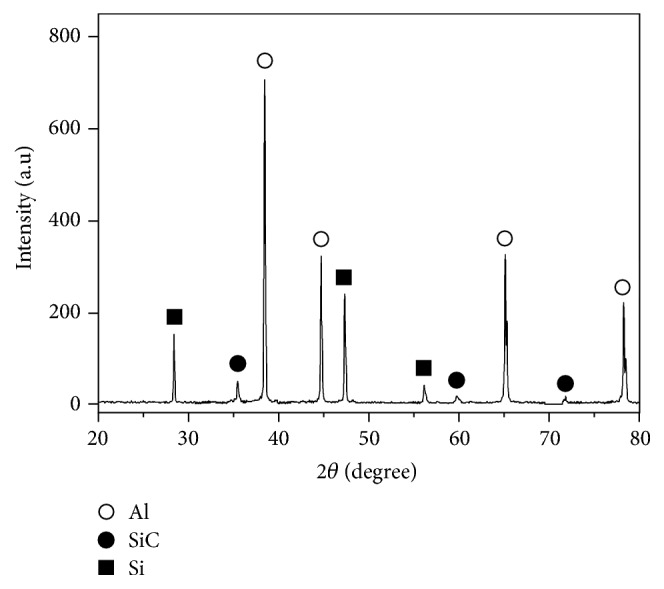
XRD pattern of the SiC/Al composites with Si/C mass ratio of 5 : 1.

**Figure 4:**
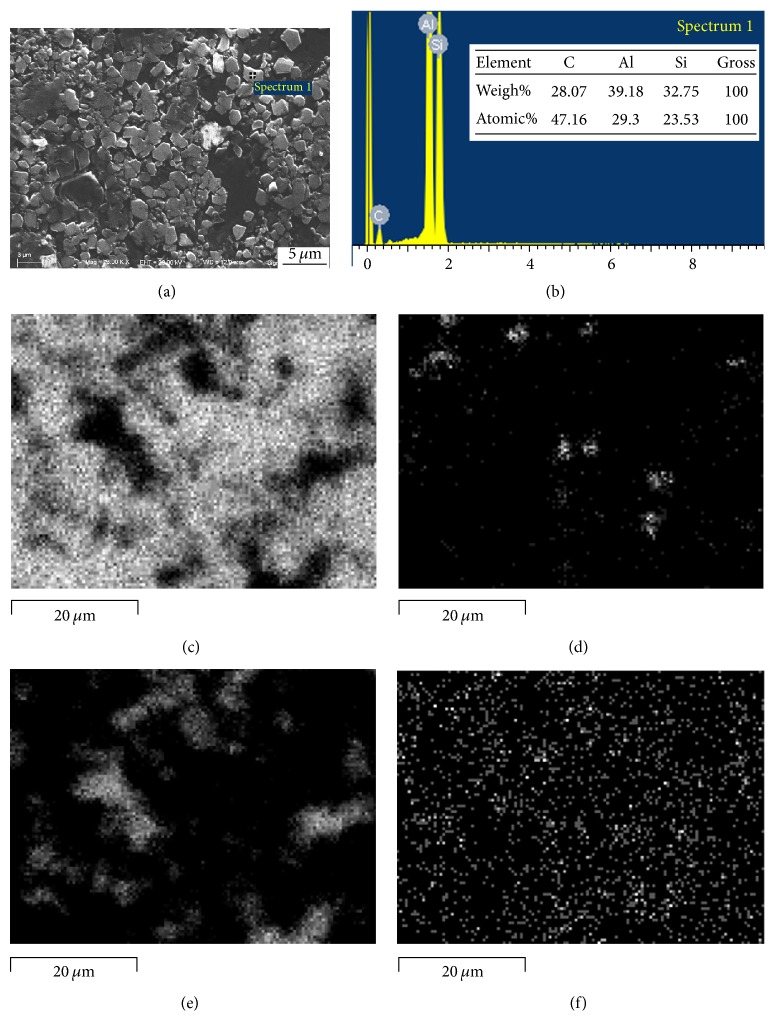
(a) SEM images of the etched surfaces of the SiC/Al composites with Si/C mass ratio of 5 : 1, (b) EDS analysis of the SiC particle as indicated in (a), and element mappings of (c) Al, (d) O, (e) Si, and (f) C.

The in situ SiC/Al composite was firstly fabricated in an Al-Si-C system with the Si/C mass ratio of 5 : 1 at 950°C and holding time for 15 min.  Figure 3 shows the XRD pattern of the fabricated SiC/Al composite. It can be seen that the in situ SiC/Al composite was successfully fabricated and no Al_4_C_3_ phase existed in the composite.  Figure 4(a) shows the SEM images of the etched surfaces of the fabricated SiC/Al composite. It can be observed that a large quantity of irregular blocky-shaped particles is distributed in the Al matrix. By the EDS analysis of the content of Si and C elements in (Figure 4(b)), the irregular blocky-shaped particles are identified as SiC. According to Du et al. reports [28], it can be known that the formation of SiC is achieved via the replacement reaction of Si and Al_4_C_3_. Hence the obtained SiC particles are liable to inherit morphological characteristics of the reactant of Al_4_C_3_. Figures 4(c)–4(f) show the element mapping of the SiC/Al composite. It can be seen that there are bulk crystal Si and SiC particles, and they are nearly uniformly distributed in the Al matrix. Meanwhile, a little amount of oxygen can be found, because the raw materials contained trace amounts of oxygen. Finally, the above results indicate that the pure SiC/Al composites are synthesized by in situ method at 950°C and holding time for 15 min by combustion synthesis and hot press consolidation.

### 3.2. Effect of the Si/C Mass Ratio

In order to investigate the effect of Si/C mass ratio on the phase constitution and microstructure of the in situ SiC/Al composites, the SiC/Al composites with different the Si/C mass ratio were fabricated.

**Figure 5:**
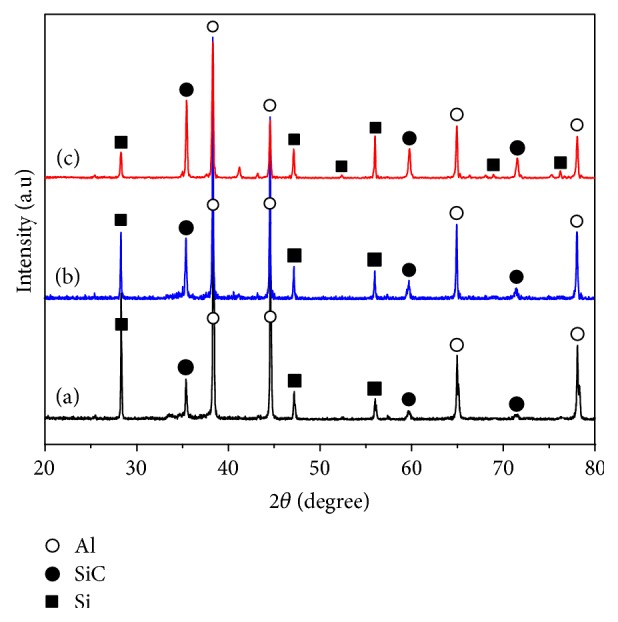
XRD patterns of the SiC/Al composites with different Si/C mass ratio: (a) Si/C mass ratio = 4 : 1, (b) Si/C mass ratio = 5 : 1, and (c) Si/C mass ratio = 6 : 1.

**Figure 6:**
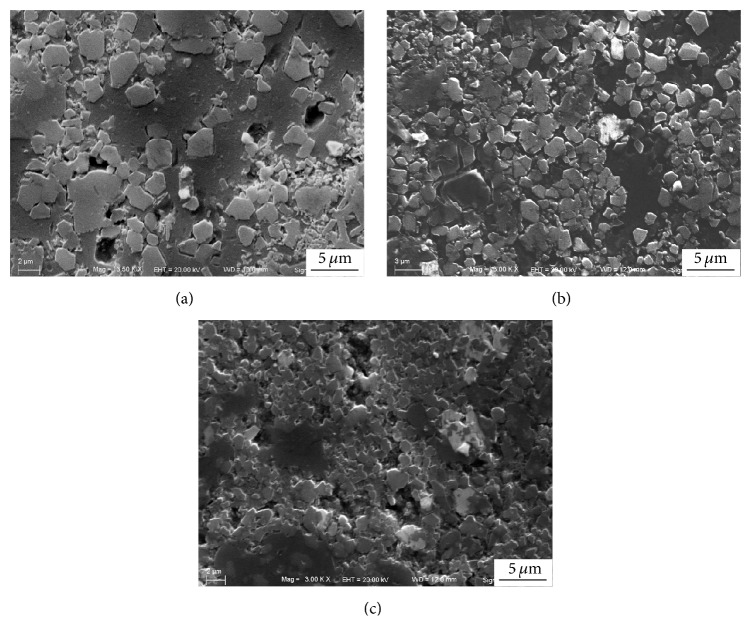
SEM images of the etched surfaces of the SiC/Al composites with different Si/C mass ratios: (a) Si/C mass ratio = 4 : 1, (b) Si/C mass ratio = 5 : 1, and (c) Si/C mass ratio = 6 : 1.

Figure 5 shows the XRD patterns of the SiC/Al composites with different Si/C mass ratio (4 : 1, 5 : 1, and 6 : 1). It can be seen that the main phases are Al, Si, and SiC in these samples. The peak intensity of the SiC phase is enhanced with increasing of the Si/C mass ratio.  Figure 6 shows the SEM images of etched surfaces of the SiC/Al composites with different Si/C mass ratios. It clearly reveals that the irregular blocky-shaped SiC particles are formed in these samples. Nevertheless, the amount of SiC particles increases and the size decreases with the increase of Si/C mass ratio.

**Figure 7:**
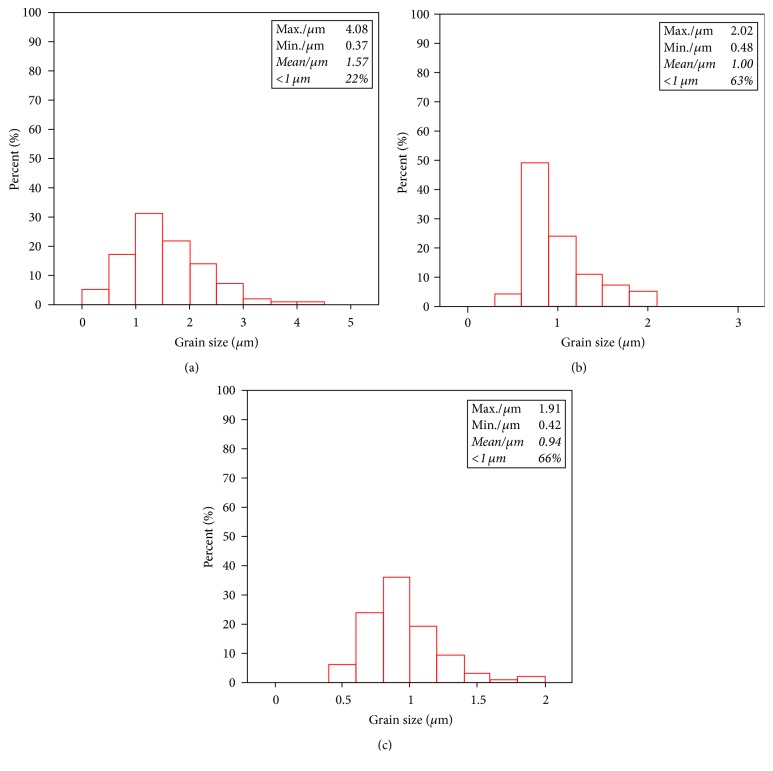
Size distribution of the SiC particles in the SiC/Al composites with different Si/C mass ratios: (a) Si/C mass ratio = 4 : 1, (b) Si/C mass ratio = 5 : 1, and (c) Si/C mass ratio = 6 : 1.

Figure 7 shows the corresponding size distribution of the in situ synthesized SiC particles in the tested samples. As shown in Figure 7(a), the maximum and average value of the SiC particles size in the composites with the Si/C mass ratio of 4 : 1 are about 4.1 *μ*m and 1.6 *μ*m, respectively, and the percentage of the submicroparticle is about 22% in total. As shown in Figure 7(b), when the Si/C mass ratio is 5 : 1, the maximum and average value of the SiC particles size are reduced to be 2.0 *μ*m and 1 *μ*m, respectively. Meanwhile, the percentage of submicroparticles increases to about 63%. As shown in Figure 7(c), when the Si/C mass ratio is 6 : 1, the maximum and average value of the SiC particle size are reduced to be 1.9 *μ*m and 0.9 *μ*m, respectively. The percentage of submicroparticles increases up to about 66%. The results indicate that the increase of the Si/C mass ratio can reduce the size of the SiC particles and lead to more uniform distribution of the particle size. Particularly, when the Si/C mass ratio increases from 4 : 1 to 5 : 1, the size of SiC particles is reduced significantly. The reason is that when the concentration of Si in the system increases, the contact opportunity between Al_4_C_3_ phase and Si atoms increases; consequently SiC nucleation rate increases. The average hardness value of the SiC/Al composites with different Si/C mass ratio (4 : 1, 5 : 1, 6 : 1) is 65.3 HB, 73.8 HB, and 75.6 HB, respectively. It can be found that when the percentage of the SiC submicroparticle in the composites increases from 22% to 63%, the hardness is increased by 13%.

The above results indicate that the increase of the Si/C mass ratio is helpful to synthesis of SiC particles. However, when the remaining Si in Al matrix is much more than the eutectic composition (12.6%) after in situ synthesized SiC, the additional Si leads to the rapid reduction of the compactness, strength, and ductility of the sample [33]. As a result, Si content is about 12% in research on most Al-Si alloy [34–36]. Therefore, the Si/C mass ratio of 5 : 1 is considered to be reasonable for the fabrication of the in situ SiC/Al composites.

### 3.3. Effect of the Holding Time

**Figure 8:**
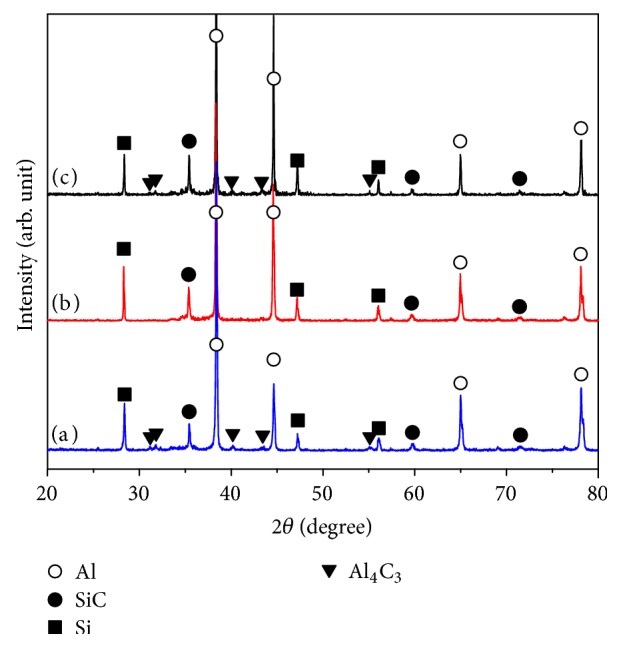
XRD patterns of the SiC/Al composites with different holding times: (a) 0 min, (b) 15 min, and (c) 30 min.

The holding time in the fabrication process of the in situ SiC/Al composites is the dynamic factor of direct impact on the reaction process; therefore the SiC/Al composites with the Si/C mass ratio of 5 : 1 under different holding time (0, 15, 30 min) were fabricated, respectively.  Figure 8 shows the XRD patterns of the SiC/Al composites with different holding time (0, 15, and 30 min). It can be seen that the products in the sample with the holding time of 15 min are mainly Al, Si, and SiC phases, shown in Figure 8(b). Meanwhile, the Al_4_C_3_ phases appear in the sample without holding and with the holding time of 30 min as shown in Figure 8((a) and (c)).

In the Al-Si-C systems, the synthesis reaction of SiC is mainly limited by the diffusion of Si atoms. If without holding in the fabrication, the first generated Al_4_C_3_ phases have not enough time to react completely with Si atoms. Meanwhile, when the holding time is extended to 30 min, the Si concentration in the system is decreased with the continual reaction between Al_4_C_3_ and Si atoms. The above two factors will lead the remaining of Al_4_C_3_ phases to appear in composites.

**Figure 9:**
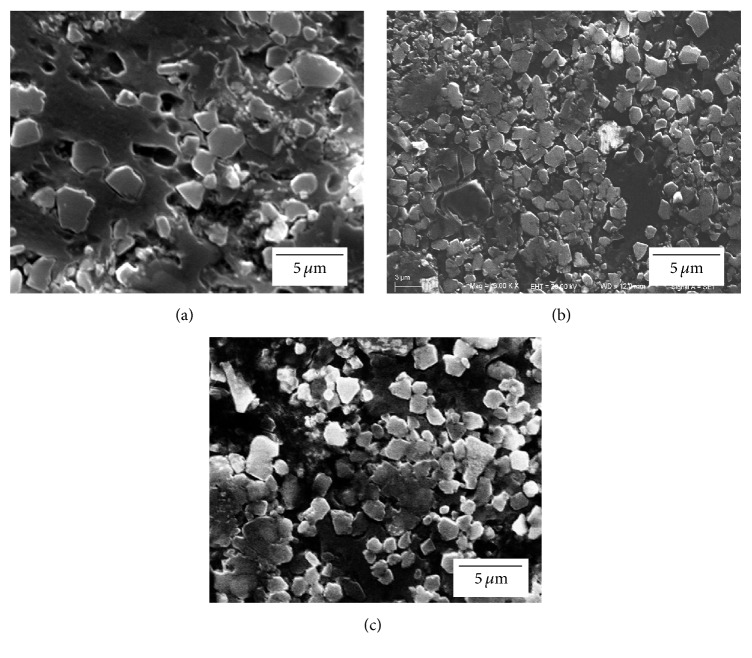
SEM images of the etched surfaces of the SiC/Al composites with different holding times: (a) 0 min, (b) 15 min, and (c) 30 min.

**Figure 10:**
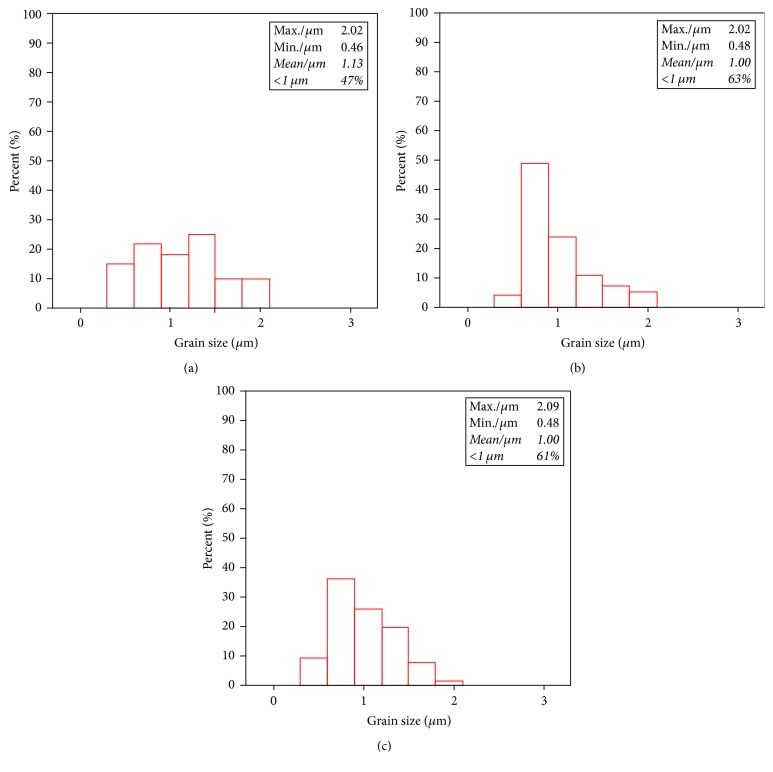
Size distribution of the SiC particles in the SiC/Al composites with different holding times: (a) 0 min, (b) 15 min, and (c) 30 min.

Figure 9 shows the SEM images of etched surfaces of the SiC/Al composites with different holding time (0, 15, and 30 min). It can be seen that the in situ SiC particles with the irregular blocky-shape formed in the three samples. Nevertheless, SiC particles are few when the holding time is 0 min. With the holding time of 15 min, the amount of SiC particles is increased. Meanwhile, with further increase of the holding time (30 min), the amount of the SiC particles has no obvious changes.  Figure 10 shows the corresponding size distribution of the in situ synthesized SiC particles in the tested samples under different holding times. It can be seen that holding times had no discernible effect on the SiC particles size. Nevertheless, the average hardness value of the SiC/Al composites with different holding time (0 min, 15 min, and 30 min) is measured to be 58.5 HB, 73.8 HB, and 71.6 HB, respectively. It can be found that when holding time is 15 min the composites have the largest hardness value. Particularly, if there is no holding time during fabrication process, the hardness value of the SiC/Al composites is decreased by 21% because the SiC particles are few in this sample.

In general, the Al_4_C_3_ phase plays a detrimental role in the mechanical properties of the composites [37]. Therefore, it is important to strictly control the residue of Al_4_C_3_ phase in the aluminum matrix composite. According to above results, the holding time should be chosen as 15 min for the fabrication of in situ SiC/Al composites.

## 4. Conclusions

In this study, the in situ SiC/Al composites are successfully fabricated by the method of combustion synthesis and hot press consolidation. With the increase of the Si/C mass ratio, the size distribution of SiC particles becomes more uniform. When the Si/C mass ratio increases from 4 : 1 to 5 : 1, the maximum size of SiC particles was reduced from 4.1 *μ*m to 2.0 *μ*m. Meanwhile, the percentage of the submicroparticles was increased from 22% to 63% and the hardness value was increased by 13%. In addition, without holding and with the holding time of 30 min in the fabrication process of the in situ SiC/Al composites, the transition phase Al_4_C_3_ was residue. Furthermore if there is no holding time the SiC particles were few in composites; as a result the average hardness value was decreased by 21%. When the holding time was set to be fifteen minutes, the Al_4_C_3_ phase totally reacts with Si atoms to form SiC particles, and the average hardness value was 73.8 HB.

## Figures and Tables

**Table 1:** Characteristics of the raw materials used in experiment.

Raw materials	Purity (wt.%)	Particle size	Morphology	Production units
Al	≥99.7	<48 *μ*m mesh	Spherical particles	Shanghai ST-Nano Science and Technology Co., Ltd.
Si	≥99.9	<48 *μ*m mesh	Irregularly shaped particles	
C-black	≥99.9	100 ± 20 nm	Spherical particles	

**Table 2:** Weight of the raw materials with different Si/C mass ratio used in the fabrication procedure.

Si/C mass ratio	Al	Si	C
4 : 1	75 g	20 g	5 g
5 : 1	70 g	25 g	5 g
6 : 1	65 g	30 g	5 g
